# Deciphering the Interaction between *Coniella granati* and Pomegranate Fruit Employing Transcriptomics

**DOI:** 10.3390/life14060752

**Published:** 2024-06-13

**Authors:** Athanasios Tsafouros, Polina C. Tsalgatidou, Anastasia Boutsika, Costas Delis, Annamaria Mincuzzi, Antonio Ippolito, Antonios Zambounis

**Affiliations:** 1 Department of Agriculture, University of the Peloponnese, 24100 Kalamata, Greece; 2 Hellenic Agricultural Organization-DIMITRA (ELGO-DIMITRA), Institute of Plant Breeding and Genetic Resources, 57001 Thessaloniki, Greece; 3 Department of Soil, Plant and Food Sciences, University of Bari Aldo Moro, 70126 Bari, Italy

**Keywords:** defense responses, immunity, plant–microbe interactions, susceptibility

## Abstract

Pomegranate fruit dry rot is caused by *Coniella granati*, also referred as *Pilidiella granati*. In order to decipher the induced responses of mature pomegranates inoculated with the pathogen, an RNA-seq analysis was employed. A high number of differentially expressed genes (DEGs) were observed through a three-time series inoculation period. The transcriptional reprogramming was time-dependent, whereas the majority of DEGs were suppressed and the expression patterns of specific genes may facilitate the pathogen colonization at 1 day after inoculation (dai). In contrast, at 2 dai and mainly thereafter at 3 dai, defense responses were partially triggered in delay. Particularly, DEGs were mainly upregulated at the latest time point. Among them, specific DEGs involved in cell wall modification and degradation processes, pathogen recognition and signaling transduction cascades, activation of specific defense and metabolite biosynthesis-related genes, as well in induction of particular families of transcriptional factors, may constitute crucial components of a defense recruiting strategy employed by pomegranate fruit upon *C. granati* challenge. Overall, our findings provide novel insights to the compatible interaction of pomegranates—*C. granati* and lay the foundations for establishing integrated pest management (IPM) strategies involving advanced approaches, such as gene editing or molecular breeding programs for disease resistance, according to European Union (EU) goals.

## 1. Introduction

Pomegranate (*Punica granatum* L.) cultivation is practiced from ancient times and nowadays is rapidly extending [1,2]. Pomegranates are considered functional foods due to their high polyphenol content, leading to a growing demand for fresh pomegranates in recent years [2]. However, physiological disorders and fungal infections lead to the postharvest decline in fruit amount and quality, potentially jeopardizing pomegranate marketability [3].

The fungus *Coniella granati* (syn. *Pilidiella granati*) has been pinpointed as one of the leading causal agents of post-harvest decay in pomegranates, impairing the market value of the product [4]. Particularly, this pathogen causes plant and fruit symptoms, such as collar rot, leaf spot, and fruit decay resulting in significant post-harvest losses that may range up to 30% [1]. However, despite its importance, information about its epidemiology [1] and host–plant interactions is scarce.

In the case of fungal infections, a finely tuned series of defense responses are induced by plants. Effectors release and pathogen-associated molecular patterns (PAMPs) contribute to pathogen recognition through pattern recognition receptors (PRR) and lead to pathogen-triggered immunity (PTI) [5]. In turn, PTI may activate the release of reactive oxygen species (ROS) and subsequently a defensive machinery comprising of a plethora of cell wall modifications, and molecular and biochemical changes in hosts. The induced defense mechanism against many necrotrophic and biotrophic pathogens also includes various non-enzymatic components, such as the production and accumulation of phenolic compounds and phytoalexins [6]. Particularly, phenolic compounds are reported to have either direct antifungal activities or are able to trigger signal transduction pathways that activate plant defense mechanisms leading to the induction of secondary metabolism pathways [1,7,8]. Recently, the polyphenol content of pomegranate fruit is positively associated with tolerance to *C. granati,* exhibiting a protective effect against infection [1,9]. Furthermore, the activation of the phenylpropanoid pathway by enhanced induction of specific genes related to this pathway along with the production of ROS, were putatively involved in the lower susceptibility of the ‘Wonderful’ cultivar compared to the more sensitive ‘Mollar de Elche’ [1].

In order to obtain valuable insights into the interactions between plants and pathogens, RNA-sequencing (RNA-seq) technology has been widely adopted [10]. This technology allows for the accurate capture of host transcriptome responses during infections. As a result, hundreds of transcriptome profiling studies have been carried out to date, proving that RNA-seq is an advanced method to decipher the molecular mechanism underlying these interactions [11]. Simultaneously, these approaches would aid in the continuous identification of new resistance genes, which is crucial in setting the stage for upcoming breeding programs and improving resistance to biotic stresses [12,13]. For instance, 186 R2R3-MYB transcription factors (TFs), identified from pomegranate genome, are involved in regulating plant development, metabolite accumulation, and defense responses; similar occurs for *U-box* gene family entailed in abiotic stresses [14,15]. Nowadays, by employing state of the art next generation sequencing (NGS) platforms, such as Illumina and Single-molecule real-time (SMRT), sequencing, costs are declining and RNA-seq technology is becoming more practical [16,17]. However, although such approaches were recently employed on fruits and petals of other deciduous trees in order to decipher the defense responses against fungal infections [18,19,20], there is still a research gap in transcriptional profiles of pomegranate fruits upon their challenge with *C. granati*.

Thus, the present study aims to elucidate the transcriptomic profiles at three different time points after inoculation of *C. granati* on pomegranates. To our knowledge, this is the first report of a large-scale transcriptome analysis during the interaction between this pathogen and pomegranate fruit. Being a minor crop, any pomegranate molecular statements are marginal, making difficult research advancements; investigation of genes entailed in disease resistance mechanisms could represent the starting point for recruiting integrated pest management (IPM) strategies providing ecofriendly control means and addressing EU goals. Our novel findings will allow us to elucidate the complex processes underlying pomegranate susceptibility and the defense responses that are triggered on fruits during the interaction with the pathogen.

## 2. Materials and Methods

### 2.1. Pomegranates, Pathogen Inoculation, and Physiological Indices

Healthy and mature pomegranates of cultivar Wonderful were collected from an orchard in Larissa province (central Greece). All fruits used in the trials were surface-sterilized as described previously [18] and air-dried at room temperature. The *C. granati* strain Ph1 that was previously morphologically and molecularly characterized [21] was used as inoculum. The fruits inoculation was performed as described in [1] with some minor modifications. Fruits were wounded in the central adaxial surface and a mycelial plug (2.5 mm diameter) of a 10-day old *C. granati* culture was placed into the wounding area (CG treatment); fruits inoculated by sterile potato dextrose agar (PDA) plugs were used as mock-inoculated control treatment (CT). The fruits were arranged in sealed transparent containers with high relative humidity (80%), at 26 ± 1 °C, and in the dark. To collect fruit tissues, three independent experiments were conducted for each of the two treatments, cutting sections with a sterile scalpel of the fruits peel (1 × 1 cm and 4 mm deep around the inoculation sites at 1, 2, and 3 dai. Ten fruits were used to create pooled samples for each treatment. Each sample was powdered in liquid nitrogen and then stored at −80 °C until use. Determination of physiological indices (lipid peroxidation and hydrogen peroxide assays, as well assignment of phenolic and flavonoids content) on fruits was conducted as previously described [18]. To evaluate fungal growth on pomegranates and fruits’ physiological changes, a one-way ANOVA was conducted followed by Tukey’s multiple test, to compare mean values among treatments. Data were statistically analyzed using IBM SPSS Statistics (v.25.0) (IBM Corp., Armonk, NY, USA).

### 2.2. Transcriptome Sequencing, Mapping, and Bioinformatics Analysis

Total RNA was extracted from 100 mg of powdered tissue employing the Plant/Fungi Total RNA Purification Kit (Norgen Biotek Corp., Thorold, ON, Canada) as described in [1]. To eliminate any DNA contamination, the total RNA from all samples were treated with the Ambion^®^ DNA-free™ DNase Treatment and Removal Reagents kit (Thermo Fisher Scientific, Waltham, MA, USA), according to manufacturer’s recommendations. Then, each RNA sample (200 ng) for the two treatments across the three time points was used for building up sequencing libraries (18 in their number) with the PT042 NGS RNA Library Prep Set (Novogene Ltd., Cambridge, UK). RNA-seq was performed with the Illumina Novaseq 6000 platform, generating 2 × 150 bp paired-end (PE) reads. The reference genomic assembly of pomegranate (ASM765513v2) and its gene models were used for mapping the clean PE reads using the HISAT2 software (v2.0.5) [22]. This software is a more efficient aligner tool for mapping NGS reads against a genome. DEGs were assigned using the DESeq2 R package (1.20.0) with an adjusted *p*-value ≤ 0.05, based on an absolute value of log2foldchange ≥ 1 [23]. This method provides statistical routines for determining differential expression in digital gene expression data using a model based on the negative binomial distribution; the resulting *p*-values were adjusted for multiple testing using the procedure of Benjamini and Hochberg for controlling the false discovery rate. Using the clusterProfiler R package (3.8.1), an enrichment analysis of Gene Ontology (GO) terms was conducted and GO terms with a *p*-value < 0.05 were designated as significantly enriched [12]. This package automates the process of GO terms classification and the enrichment analysis of gene clusters using combined analysis and visualization modules. As previously performed [24], DEGs were also assigned to KEGG Orthology enriched terms. KEGG (Kyoto Encyclopedia of Genes and Genomes) is a database resource for understanding high-level functions and utilities of the biological pathways from large-scale molecular datasets.

### 2.3. Validation of DEGs by RT-qPCR

To validate the RNA-seq data, relative gene expression of nine, randomly selected, pomegranate DEGs was conducted through real-time quantitative PCR (RT-qPCR) analysis, following the procedure as previously described [25]. PCR reactions were conducted in triplicate and the expression patterns were compared to the constitutively expressed housekeeping gene *EF-1α* using the specific primers previously described [1]. The relative quantitative expression ratios of the inoculated samples relative to the corresponding controls were tracked using the 2^−△△CT^ method [26]. This method allows analyzing data from real-time, quantitative PCR experiments in order to capture the relative quantification of target genes.

## 3. Results

### 3.1. Coniella granati Inoculation on Pomegranate Fruit and Symptoms

**Figure 1:**
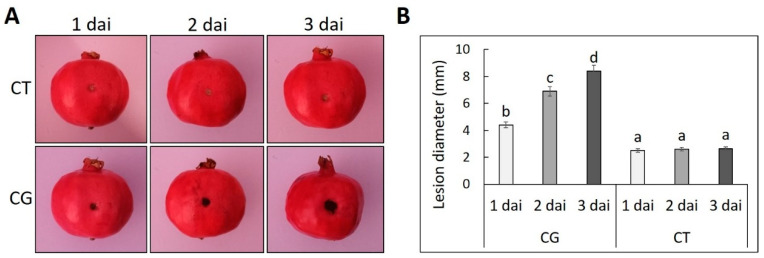
Pomegranates (cv. Wonderful) inoculated with *C. granati* (CG) at 1, 2, and 3 days after inoculation (dai). Untreated pomegranates were used as a control (CT). (**A**) Visual symptoms. (**B**) Mean lesion diameter (mm). Different letters indicate differences between treatments (*p* < 0.01).

The first visual symptoms of fungus infection in the inoculated wounds were observed at 1 dai (Figure 1A) with a mean lesion diameter approximately at 4.7± 0.3 mm. From this time point and thereafter, the mean lesion diameter was progressively increased at the CG treatments reaching its peak (8.4 ± 0.4 mm) at 3 dai (Figure 1B).

### 3.2. Physiological Changes on Pomegranate Fruit in Response to Coniella granati Infection

Total flavonoid and phenolic amount were significantly increased upon *C. granati* inoculation (CG treatment) compared to the mock-inoculated fruits (CT treatment), as shown in Table 1. Total flavonoids reached their peak level at 3 dai, whereas the phenolic compounds decreased progressively over the inoculation period, reaching their lowest level at 3 dai. Although total flavonoids in the CT treatment significantly increased over time, the level of total phenolics remained unaffected over the three time-series period. Furthermore, both TBARS (lipid peroxidation) and H_2_O_2_ levels in the CG treatment were significantly higher compared to CT treatment at all time points (Table 1). The amount of both TBARS and H_2_O_2_ levels increased over time, reaching their highest level at 3 dai in the CG treatment.

### 3.3. RNA-seq Analysis

**Figure 2:**
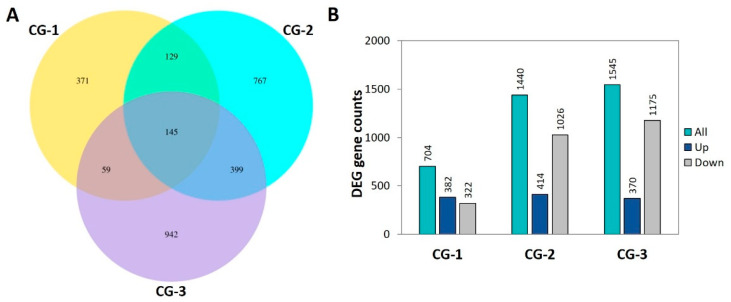
Differentially expressed genes (DEGs) between *C. granati* inoculated (CG) pomegranates (cv. Wonderful) at 1, 2, and 3 days after inoculation (dai). (**A**) Venn diagram illustrating DEGs commonly regulated across the three comparison groups (CG-1, CG-2, and CG-3). (**B**) Number of total (all), up- and downregulated DEGs among the three comparison groups (CG-1, CG-2, and CG-3).

In order to decipher the transcriptomic changes that occurred on fruits over the three time points upon the pathogen inoculation, dual RNA-seq analysis was performed using fruit samples inoculated with the fungus (CG) or mock-inoculated (CT). Thus, the transcriptome analysis was performed on 18 fruit samples employing three biological replicates for each treatment at 1, 2, and 3 dai. Approximately 87% of the totaling 651,972,806 high-quality PE reads were uniquely mapped to the reference pomegranate genomic assembly (Table S1). Three comparison groups, namely CG-1, CG-2, and CG-3 that corresponded to 1, 2, and 3 dai, respectively, were assigned to track the transcriptional responses of fruits by comparing the pairwise expression profiles between the CG and CT treatments (Table S2). In total, there were identified 3,689 DEGs. Particularly, there were detected 704, 1440, and 1545 DEGs in the CG-1, CG-2, and CG-3 comparison groups, respectively (Figure 2A). The increasing trend in the total number of DEGs is primarily attributed to the progressively increasing number of upregulated DEGs from the 1 up to 3 dai. Additionally, at both 2 and 3 dai (CG-2 and CG-3 comparison groups), the number of upregulated DEGs exceeded that of the downregulated ones, whereas the opposite was observed in the 1 dai (CG-1 comparison group). Among the identified DEGs, 371, 767, and 942 were exclusively detected in the CG-1, CG-2, and CG-3 comparison groups, respectively, while 145 DEGs were constitutively expressed in the three groups (Figure 2B).

### 3.4. Functional Annotations and Classifications of DEGs

**Figure 3:**
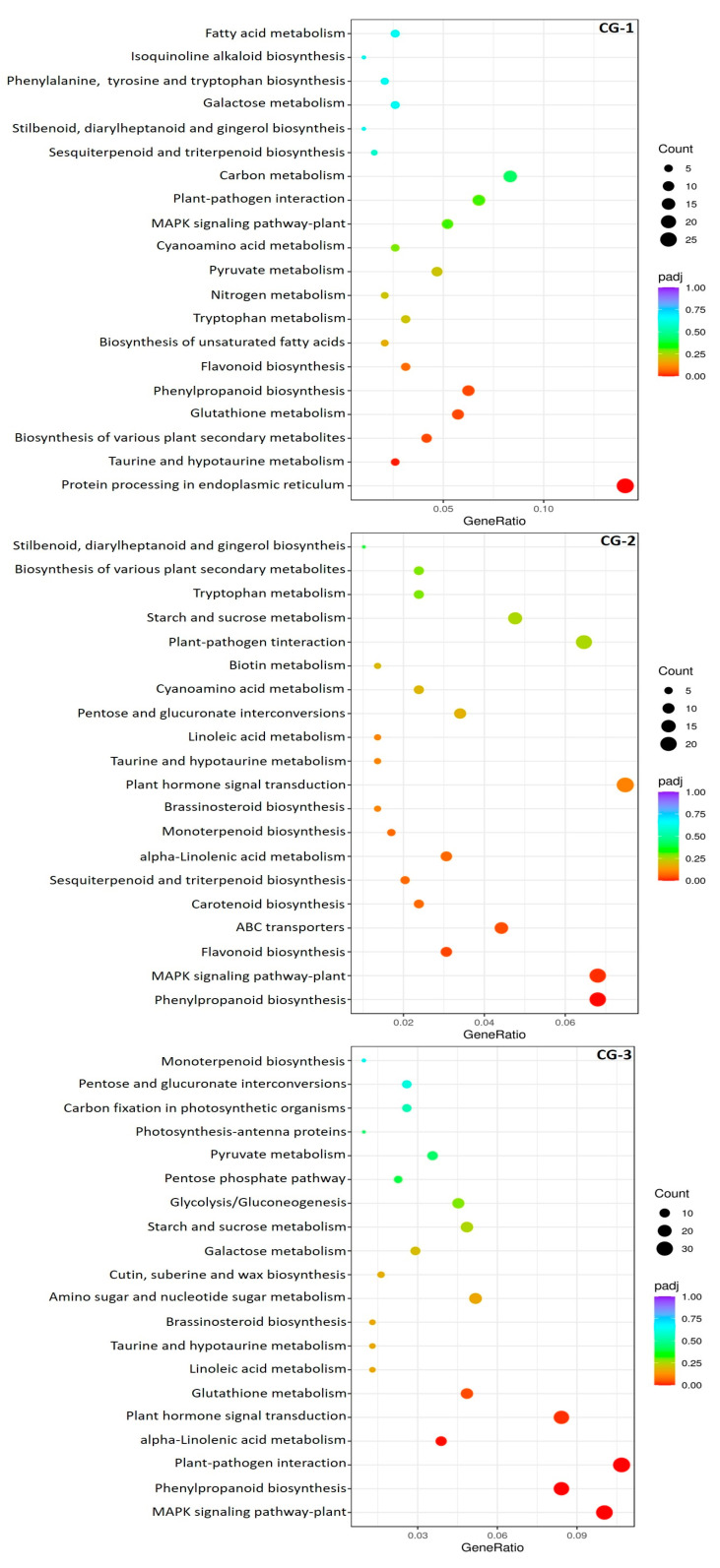
KEGG pathway enrichment scatter-plot from DEG analysis on pomegranates (cv. Wonderful) inoculated with *C. granati* (CG) in comparison to control treatment (CT) at 1, 2, and 3 days after inoculation (dai) across the three comparison groups (CG-1, CG-2, and CG-3). DEG counts being annotated in the corresponding pathways are depicted, whereas the significant size (adjusted *p*-value, padj) of the enrichment is indicated by a color.

In terms of biological processes, GO terms of ‘defense response’ and ‘response to stress’ exhibited significant enrichment throughout the three-day period. Regarding the cellular components, GO terms of ‘extracellular region’, ‘cell wall’, and ‘apoplast’ were enriched at 3 dai. The terms ‘transcription regulator activity’ and ‘DNA-binding transcription factor activity’ were significantly enriched at 2 and 3 dai in terms of molecular functions (Figure S1). The classification of DEGs in KEGG pathways revealed a significant enrichment of ‘phenylpropanoid biosynthesis’ pathway at 1 dai. This pathway remained enriched, as well as the ‘MAPK signaling pathway’, ‘alpha-linolenic acid metabolism’, ‘plant hormone signal transduction’, and ‘plant-pathogen interaction’ pathways both at 2 and 3 dai (Figure 3).

### 3.5. DEGs Transcriptional Profiles

**Figure 4:**
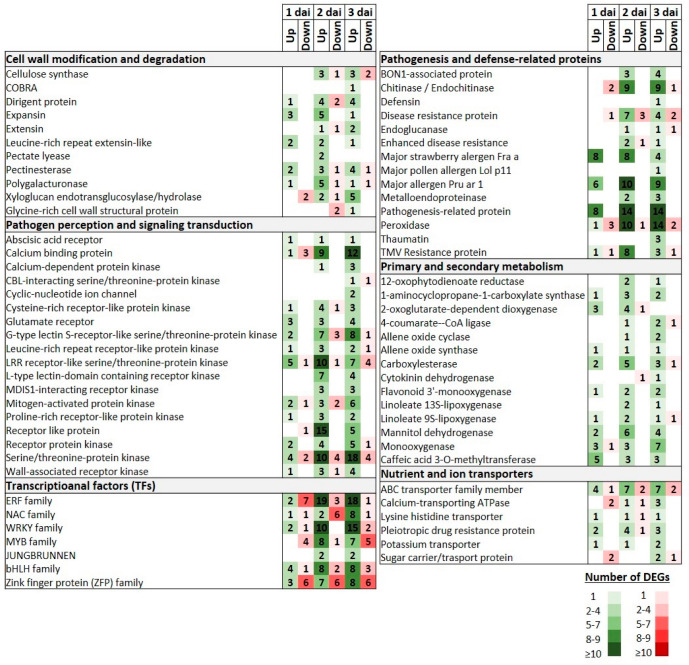
Heat map of selected upregulated (Up) and downregulated (Down) key DEGs in pomegranates (cv. Wonderful) inoculated with *C. granati* (CG) in comparison to control treatment (CT) at 1, 2, and 3 days after inoculation (dai). The numbers of the differentially expressed transcripts are reported for both downregulated (red color scale) and upregulated (green color scale) gene groups at 1, 2, and 3 dai that correspond to CG-1, CG-2, and CG-3 comparison groups, respectively.

DEGs associated with cell wall modification and degradation processes were induced across the inoculation period, whereas a predominant upregulation was observed, particularly at 3 dai. Thus, the induction patterns of DEGs encoding COBRA (COBL), extensin (EXT), xyloglucan endotransglucosylase/hydrolase (XTH), glycine-rich cell wall structural protein (GRP) proteins, as well as four dirigent (DIR) protein homologues were constitutively activated at the last time point. Notably, expansin (EXP), pectate lyase (PL), and polygalacturonase (PG) encoding DEGs were mainly upregulated at two dai, whereas a similar activation, albeit to a lesser extent, was also unraveled at one dai. Finally, throughout the three time points, pectinesterase (PME) encoding DEGs were primarily upregulated (Figure 4; Table S3).

Many DEGs, including different kinds of receptor-like kinases (RLKs) and receptor-like proteins (RLPs), encoding pathogen recognition receptors (PRRs), along with DEGs involved in downstream defense-related signaling transduction were significantly upregulated both at 2 and 3 dai. Thus, several DEGs encoding calcium-binding protein (CBP), calcium-dependent protein kinase (CDPK), cysteine-rich receptor-like protein kinase (CRK), L-type lectin-domain-containing receptor kinase (L-type LecRLK), G-type lectin S-receptor-like serine/threonine-protein kinase (GsSRK), glutamate receptor (GR), proline-rich receptor protein kinase (PERK), mitogen-activated protein kinase (MAPK), receptor protein kinase (RK), and wall-associated receptor kinase (WAK), along with various RLPs exhibited substantial upregulation at 2 dai and thereafter. Similarly, the expression profiles of DEGs encoding serine/threonine protein kinase (STPK) showed a significant activation of those genes mainly at 3 dai (Figure 4; Table S4).

A few TFs families were induced with a rather consistent trend through the 3 day inoculation period. Thus, for example, even though the early downregulation at 1 dai of a few members of the ERF family, a notable shift to upregulation was detected at 2 dai, which was sustained at a steady state at 3 dai. In the case of WRKY TFs encoding DEGs, their expression profiles demonstrated a progressively elevated activation across the three time points, and notably, no downregulated genes were detected at 2 dai. Similar expression profiles were also observed for DEGs encoding members of MYB and basic Helix-Loop-Helix (bHLH) families. In contrast, a distinct expression profile was revealed for the Zinc finger protein (ZFP) family, whereas a higher number of downregulated DEGs were detected during the initial two time points, and a reversal occurred at the 3 dai with an increased number of upregulated genes (Figure 4; Table S5).

DEGs encoding peroxidase and various pathogenesis-related proteins displayed an escalating trend for upregulation across the three time points. Chitinase and endochitinase-encoding DEGs were initially downregulated at 1 dai, followed by an upregulation at 2 and 3 dai, while DEGs encoding thaumatin and defensin proteins were induced only at 3 dai. Furthermore, DEGs encoding metalloendoproteinase (MMPs) as well BON1-associated proteins (BAPs) were constitutively upregulated only at 2 and 3 dai. Notably, various classes of major allergen proteins were constitutively upregulated across the three time points (Figure 4; Table S6).

Numerous DEGs related to metabolic processes, such as in secondary and primary metabolism, were activated with most of them demonstrating significant upregulation at 2 and 3 dai. For example, DEGs encoding caffeic acid 3-O-methyltransferase (COMT), which are related both to the phenylpropanoid biosynthesis and tryptophan metabolism were constitutively upregulated across the three time points. It is worth mentioning that similar expression profiles were observed for DEGs encoding mannitol dehydrogenase (MTD) proteins. Furthermore, DEGs encoding 12-oxophytodienoate reductase (OPR), which represents a key enzyme in the biosynthesis of jasmonic acid (JA) and the ‘alpha-linolenic acid metabolism’ pathway, exhibited a constitutive activation profile at 2 and 3 dai. This KEGG pathway that is involved in primary metabolism was also enriched at 2 and 3 dai due to the upregulation of genes encoding allene oxide cyclase (AOC), allene oxide synthase (AOS), and different types of linoleate lipoxygenase (LOX) proteins. Notably, DEGs encoding 2-oxoglutarate-dependent dioxygenase (2-OGD) were upregulated at 1 dai (Figure 4; Table S7).

Finally, DEGs encoding nutrient and ion transporters exhibited significant induction, with most of them showing mainly an upregulation at the 3 dai. Notably, ABC transporters and pleiotropic drug resistance protein-encoding DEGs demonstrated a predominant upregulation at 2 and 3 dai, with a lower activation at 1 dai (Figure 4; Table S8).

### 3.6. Validation of DEGs by RT-qPCR

To verify the RNA-seq data, a group of nine DEGs were randomly selected; the list of the gene-specific primer pairs is provided in Table S9. The expression values of the RNA-seq analysis were verified using a RT-qPCR assay. The expression profiles of all tested genes were comparable to those of the RNA-seq analysis (Figure S2).

## 4. Discussion

The precise transcriptional responses that pomegranate fruit undergo when infected with *C. granati* remain widely unknown. Therefore, the main objective of this study was to decipher the transcriptome dynamics recruited during this host–pathogen interaction. This would allow clarification of the expression profiles of fruits DEGs that contribute to the promotion of susceptibility or the activation of defense-related responses. According to our results, pomegranates were able to induce a level of basal defense to some extent, even at the earliest time point of inoculation (1 dai), which was not enough to prevent the fungal penetration and the mycelial growth. In fact, fruit tissues could initiate defense mechanisms even in the face of overwhelming fungal infections [27,28]. During the later time points, primarily at 3 dai, an extensive transcriptional reprogramming occurred that was coupled with a delayed activation of DEGs involved in processes related to strengthening and reinforcing cell wall assembly, pathogen recognition and downstream immune signaling transduction, upregulation of numerous TFs, induction of several pathogenesis and defense-related genes, as well as activation of primary and secondary metabolism. This was also amplified as ‘MAPK signaling pathway’, ‘alpha-linolenic acid metabolism’, ‘phenylpropanoid biosynthesis’, ‘plant hormone signal transduction’, and ‘plant–pathogen interaction’ pathways were significantly enriched at 3 dai. Overall, our RNA-seq data highlight a time-dependent transcriptional reprogramming upon *C. granati* inoculation with a delayed activation of defense responses. This is consistent with other surveys upon inoculation with fungal pathogens in kiwifruits, strawberries, and peach fruits [12,18,27,28]. Although most of the affected DEGs were upregulated, particularly at 3 dai, a significant portion was downregulated across all time points, mainly at 1 dai.

The cell wall of host tissues constitutes the primary structural barrier of the plant defense mechanisms in response to pathogen attack [28]. On the other hand, disassembly and degradation of the cell wall is known to increase fruit vulnerability to pathogen infection [12,28]. Our findings indicate that many DEGs associated with cell wall modification impeding the pathogen penetration by thickening, stiffening, and lignification of cell walls [12,29,30], such as those of *CesA*s, *EXT*s, *GRP*s, *DIR*s, *XTH*s, and *COBL*s genes, were differentially regulated across the inoculation time points and were mainly upregulated at 3 dai. Fruit susceptibility to pathogen infection is also amplified by ripening [12]. We speculate that *C. granati* may further stimulate the ripening process by manipulating *PG*s, *PL*s, and *EXP*s during the early infection stage, as previously reported in *B. cinerea* infections [31]. Notably, *PL*s and *EXP*s genes were upregulated at 2 dai, implying their involvement in pomegranate fruit susceptibility and cell wall extensibility. Previously, the silencing of a *PL* gene in tomato enhanced fruit firmness and reduced susceptibility to *B. cinerea*, while cellulose and hemicellulose concentrations were raised [32]. Similarly, during pathogenesis the upregulation of most *PME*s genes across all time points could lead to cell wall loosening facilitating pathogen colonization [12].

A large repertoire of RLKs and RLPs-encoding genes involved in plant–microbe interaction, pathogen recognition, and signaling were upregulated mainly at 2 and 3 dai. Previously, *RLK*s, *G-*type *LecRK*s, and *WAK*s genes were reported to be involved in defense responses to pathogens [12,18,20,25,33,34,35]. Furthermore, *CRK*s, *PERK*s, and *CDPK*s genes are related to immune signaling pathways, acting as plant defense regulators [36].

TFs such as those of ERF, WRKY, MYB, bHLH, and ZFP families play a key role in the regulation of defense mechanisms [12,18,20,37]. Regarding *C. granati* infection, a large set of TFs were induced over the inoculation period. As a result, the GO terms ‘transcription regulator activity’ and ‘DNA-binding transcription factor activity’ were significantly enriched at 2 and 3 dai. Among the TFs-encoding genes significantly upregulated mainly at 3 dai, there were members of the bHLH family described as JA-mediated transcriptional regulators [38]; ERFs represent a key regulatory hub, integrating hormone production and redox signaling in the plant response to fungal inoculation [39]. However, within the bHLH family, MYC2 which contributes to susceptibility to gray mold [40], was constitutively upregulated at all time points. On the other hand, members of the ZFP and WRKY families mediate metabolic modifications, leading to the establishment of defense responses against bacterial and fungal diseases, [36,41,42]. Notably, among the upregulated WRKY TFs at 3 dai, it was found a WRKY65 homolog contributed to enhanced resistance in *Arabidopsis* transgenic plants against *B. cinerea* and *Pseudomonas syringae* pv. *tomato* DC3000 (Pst) infection [43].

The activation of PRs and other defense-related genes, mainly at 2 and 3 dai, further indicates a delay in pomegranate immunity responses. Particularly, a high number of DEGs encoding chitinase, endochitinase, and peroxidase enzymes were mainly upregulated at the latest time point, along with a defensin and three thaumatin-encoding genes. It is worth to mentioning the constitutively upregulation at the three time points of various homologous of major allergen proteins belonging to various classes of PR-10 genes. These genes are positively involved in JA-mediated regulation of defense signaling and interact with metabolic components of flavonoid biosynthesis [12,44]. The activation of such genes also at 1 dai suggests that defense responses were also induced at the earliest time point albeit to a less extent. Furthermore, MMPs, belonging to the PR-10 family and considered as multifunctional effector components involved in pivotal regulatory roles in defense homeostasis [12,45], were constitutively upregulated at 2 and 3 dai. It is known that an MMP protein is required for resistance against *B. cinerea* in tomato [46], whereas activation of members of the PR-10 family suggest a further enhancement of the JA-mediated transduction of defense signaling and activation of flavonoid biosynthesis [20,44]. However, at these time points, BAP genes were upregulated; these are negative regulators of immune reactions [47], indicating suppression of defense responses and promoting susceptibility as a result of pathogen expansion.

DEGs associated with the activation of secondary metabolism were mainly induced at 2 and 3 dai. Among them, *COMT*s genes are related with both the enhancement of phenylpropanoid biosynthetic pathway and tryptophan metabolism, while playing a pivotal role in the lignin biosynthetic pathway [48]. Previously, such a gene was reported to contribute positively to wheat resistance against sharp eyespot caused by the necrotrophic fungus *Rhizoctonia cerealis* [48]. Across the inoculation period, the expression profiles of MTDs were significantly upregulated. Several fungal pathogens secrete mannitol to mitigate the ROS-mediated host defenses [49]. This process is required for their pathogenicity, whereas mannitol-deficient mutants of *Alternaria alternata* have shown decreased pathogenicity on tobacco [50]. To catabolize the pathogen’s secreted mannitol, plants activate pathogen-induced MTDs to protect themselves through ROS-mediated defenses [49]. Notably, a cytokinin dehydrogenase (CKX)-encoding gene was found to be upregulated at 3 dai. These genes are related with resistance to pathogens [51], whereas a CKX gene has been assigned as the main hub gene related to the induced tolerance of pear petals against *Monilinia laxa* [20]. This finding further suggests that indeed pomegranate defense responses are induced in delay. Furthermore, DEGs encoding OPRs enzymes that are involved in biosynthesis of secondary metabolites, the significant enrichment of ‘alpha-linolenic acid metabolism’ pathway, the JA biosynthesis and plant defense responses [52,53,54], were activated at 2 and 3 dai. The primary metabolism associated ‘alpha-linolenic acid metabolism’ pathway was also enriched at 2 and 3 dai through the upregulation of AOCs, AOSs, and LOXs genes. It is worth noting that some of these genes are also involved in JA biosynthesis highlighting the hypothesis that this phytohormone plays a pivotal role in signaling transduction mediated responses [25]. Such genes were upregulated in peach and kiwifruits during compatible interactions with *M. fructicola* and *B. cinerea*, respectively [12,18].

Notably, DEGs encoding 2-OGD were upregulated at 1 dai. These genes are involved in flavonoid biosynthesis [55], as well as in various metabolic processes including this of aliphatic glucosinolate biosynthesis that participate in defense mechanisms against fungal pathogens [56]. Furthermore, DEGs encoding flavonoid 3′-monooxygenase, a key enzyme in the flavonoid biosynthetic pathway, were constitutively upregulated at all time points. Flavonoids are plant metabolites with known antifungal activities against fungi, and their biosynthetic accumulation plays pivotal role in signaling that drives plant defense responses [57]. Consistent with this, total flavonoids were recorded to increase across the three time points after inoculation with *C. granati*.

ROS generation, which triggers an oxidative burst that coordinates the hypersensitive response, may also promote pomegranate susceptibility, acting as a virulence factor that *C. granati* utilizes to manipulate defense responses in fruits. Both TBARS which count lipid peroxidation, and H_2_O_2_ levels of CG treatment were significantly higher compared to CT treatment at every time point and they were increased over time, reaching their peak at 3 dai. In our study, a *Rboh* (respiratory burst oxidase homolog) gene, which is a PAMP-induced and involved in the regulation of ROS accumulation [58], was constitutively upregulated at the three time points. Furthermore, numerous DEGs directly participated in the ROS scavenging pathway, such as those encoding the glutathione S-transferase enzyme that were highly upregulated at 3 dai.

Particularly, during the latest stages of infection, DEGs encoding nutrient and ion transporters were induced. Among them, the pathogen may have manipulated the activation of the lysine histidine transporter-encoding genes to obtain nutrients from the decaying host cells [27]. Furthermore, DEGs encoding ABC transporters, especially those members of the G family, were upregulated at 2 and 3 dai. In plants, upon pathogen challenge, these genes are involved in the transport and secretion of secondary metabolites to cope with the infection [59].

Overall, our findings allow elucidation of the transcriptional motivations of pomegranate fruits upon their challenge with *C. granati*. Despite the high number of pathways and DEG families involved, further research is necessary in order to fully uncover this host–microbe interaction, for instance, by recruiting metabolomics and proteomics analyses. It would be also interesting to broaden research by comparing the defense responses among different pomegranate cultivars. On the other hand, fast-evolving molecular biology methods could also favor research progression, finalizing the applicability of our findings by exploiting them in breeding approaches for disease resistance, gene editing, and antifungal metabolite production. In the near future, these strategies would sustainably contribute to the design of novel disease management tools for *C. granati* in pomegranates.

## 5. Conclusions

To decipher the transcriptional responses across a compatible interaction between pomegranate fruits and *C. granati*, an RNA-seq approach was used. Even though the expression profiles of specific DEGs may have facilitated the colonization of the pathogen, particularly promoting susceptibility in the earliest stage of infection, defense responses were also identified mainly at 3 dai. Thus, a series of delayed triggering defense responses were uncovered to halt to some extent the growth of pathogen. At the latest time point, even if it was infeasible to restrict fungal growth and disease progression, fruits were able to selectively activate DEGs in delay that were involved in certain cell wall modification and degradation processes, biosynthesis of specific types of primary and secondary metabolites, pathogen recognition and induction of several defense-related TFs and PRs encoding genes. Our findings may offer a comprehensive roadmap for understanding the molecular mechanisms driving this plant–microbe interaction. These may be also useful for fully understanding pathways and genes entailed in disease resistance, in order to set-up new IPM strategies, according to EU goals, even though more research is needed for reaching these targets.

## Figures and Tables

**Table 1:** Antioxidant indicators and oxidative stress response of *C. granati* inoculated (CG) and mock-inoculated (CT) pomegranates (cv. Wonderful) at 1, 2, and 3 days after inoculation (dai). Data values represent the mean values of three biological replicates ± standard deviations per treatment. Each measure is rated to the fresh weight mass (g FW).

	Days after Inoculation (dai)	Total Flavonoids (mg Rutin/g FW)	Total Phenolics (mg GAE/g FW)	Lipid Peroxidation, TBRAS (nmole/g FW)	Hydrogen Peroxide (H_2_O_2_) (μmole/g FW)
CG	1	31.36 ± 1.20 ^d^	32.57 ± 0.60 ^b^	18.09 ± 0.13 ^cd^	1.18 ± 0.06 ^a^
	2	29.24 ± 0.64 ^c^	31.48 ± 0.40 ^c^	18.10 ± 0.25 ^cd^	1.33 ± 0.04 ^ab^
	3	36.78 ± 0.50 ^e^	30.10 ± 0.51 ^c^	19.14 ± 0.39 ^d^	1.37 ± 0.06 ^ab^
CT	1	21.50 ± 0.57 ^a^	23.27 ± 0.27 ^a^	14.06 ± 0.20 ^a^	0.97 ± 0.07 ^bc^
	2	25.57 ± 0.36 ^b^	24.35 ± 0.53 ^a^	16.29 ± 0.76 ^b^	1.10 ± 0.06 ^cd^
	3	28.12 ± 0.48 ^c^	23.45 ± 0.38 ^a^	17.51 ± 0.48 ^c^	1.11 ± 0.06 ^d^

Different superscript letters in each column represent statistical differences among treatments according to Tukey’s multiple test (*p* < 0.05).

## Data Availability

The datasets generated during the current study are available in the NCBI SRA database below: https://www.ncbi.nlm.nih.gov/, PRJNA1041602, created date 17 November 2023.

## Supplementary Materials

The following supporting information can be downloaded at: https://www.mdpi.com/article/10.3390/life14060752/s1, Figure S1: Gene ontology (GO)-based functional enrichment and categorization of the most representative differentially expressed genes (DEGs) in the comparison groups CG-1, CG-2, and CG-3 across the three time points (1, 2, and 3 dai); Figure S2: Comparison of RNA-seq and RT-qPCR expression values of selected genes after *C. granati* inoculation on pomegranates at 2 dai; Table S1: Summary of pomegranate fruit RNA-seq data; Table S2: Differentially expressed gene (DEG) expression profiles in comparison groups CG-1, CG-2, and CG-3 at 1, 2 and 3 dai, respectively; Table S3: Selected differentially expressed genes (DEGs) involved in cell wall modification and degradation across the three comparison groups (CG-1, CG-2, and CG-3); Table S4: Selected differentially expressed genes (DEGs) involved in pathogen recognition and signaling transduction across the three comparison groups (CG-1, CG-2, and CG-3); Table S5: Selected differentially expressed genes (DEGs) encoding transcriptional factors across the three comparison groups (CG-1, CG-2, and CG-3); Table S6: Selected differentially expressed genes (DEGs) encoding pathogenesis and defense-related proteins across the three comparison groups (CG-1, CG-2, and CG-3); Table S7: Selected differentially expressed genes (DEGs) involved in secondary and primary metabolism across the three comparison groups (CG-1, CG-2, and CG-3); Table S8: Selected differentially expressed genes (DEGs) encoding nutrient and ion transporters across the three comparison groups (CG-1, CG-2, and CG-3); Table S9: Specific primers employed in RT-qPCR for the evaluation of RNA-seq data.
