# Supplementary material for: Deciphering the Interaction between Coniella granati and Pomegranate Fruit Employing Transcriptomics
Source: Life (Basel). 2024 Jun 13;14(6):752. doi: 10.3390/life14060752 (PMC11205003; doi:10.3390/life14060752)
Supplement: Supplementary file 1 [file life-14-00752-s001.zip › Supplementary Figures_Life_Tsafouros.pdf]

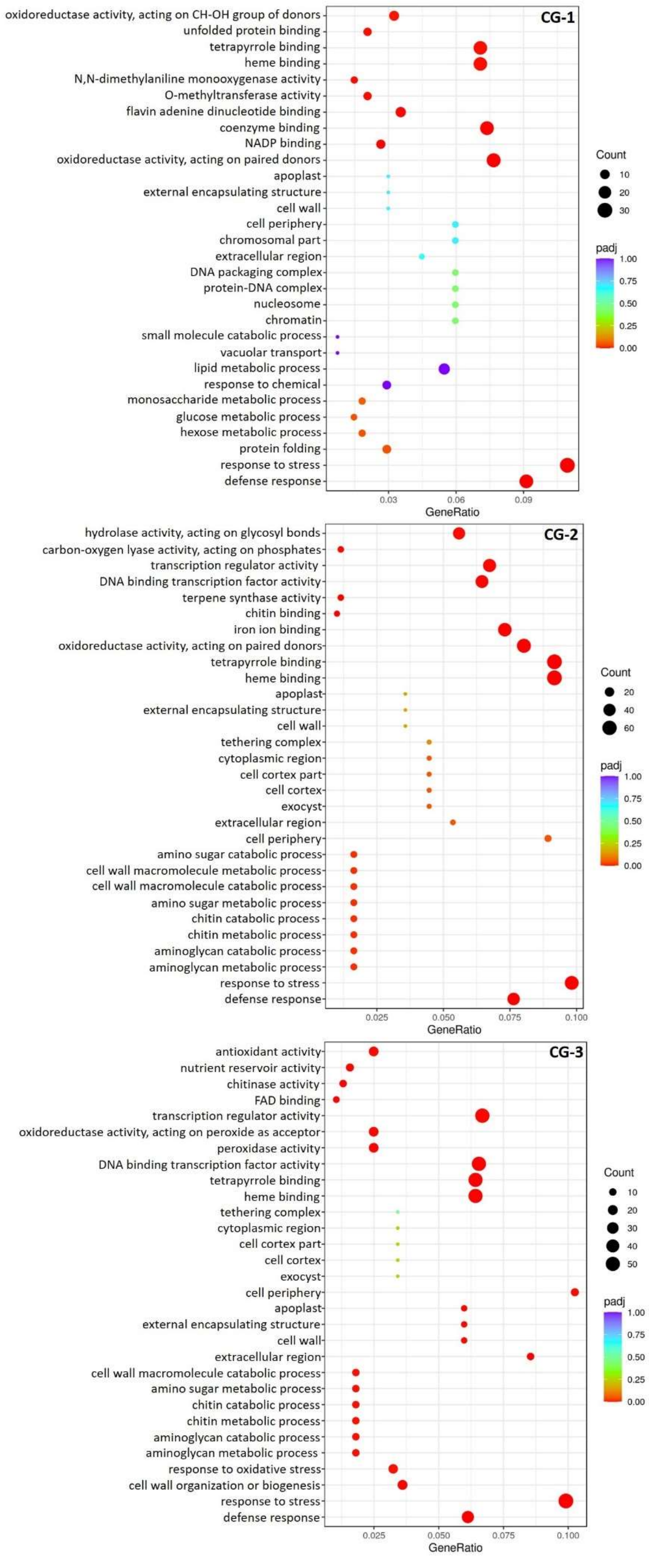

Figure S1: Gene Ontology (GO)-based functional enrichment and categorization of the most representative differentially expressed genes (DEGs) in the comparison groups CG-1, CG-2, and CG-3 across the three time points (1, 2 and 3 dai).

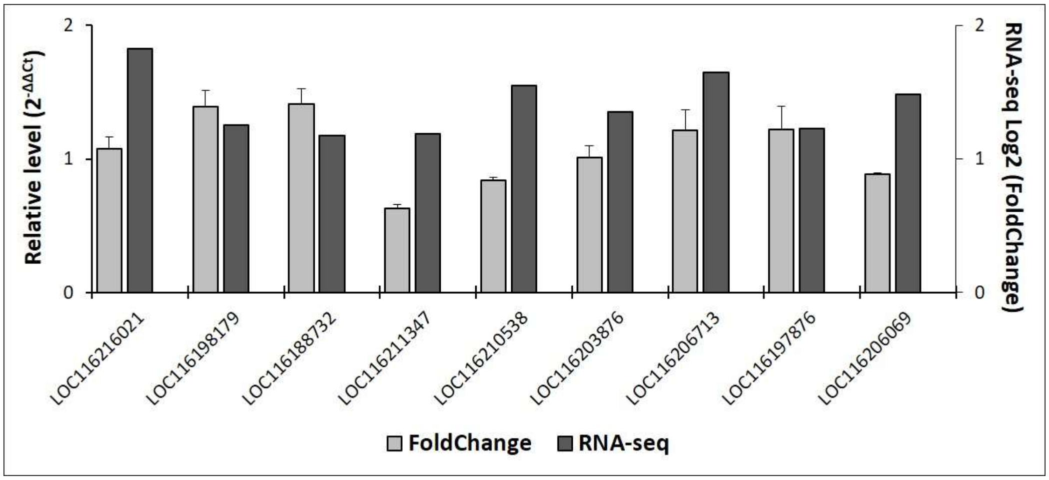

Figure S2: Comparison of RNA-seq and RT-qPCR expression values of selected genes after *C. granati* inoculation on pomegranates at 2 dai.
