# Supplementary figures and images for: Deciphering the Interaction between Coniella granati and Pomegranate Fruit Employing Transcriptomics
Source: Life (Basel). 2024 Jun 13;14(6):752. doi: 10.3390/life14060752 (PMC11205003; doi:10.3390/life14060752)

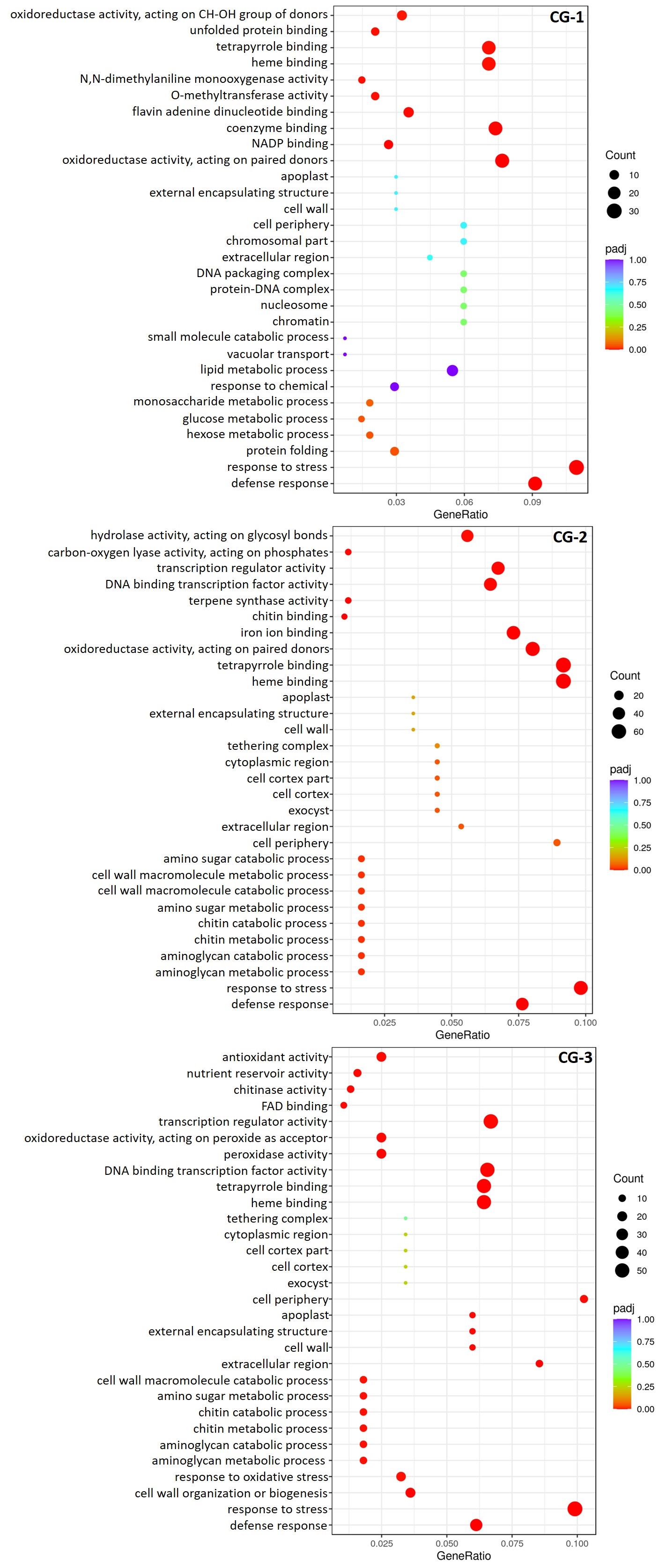

Supplement: Supplementary file 1 [file life-14-00752-s001.zip › Figure S1.jpg]

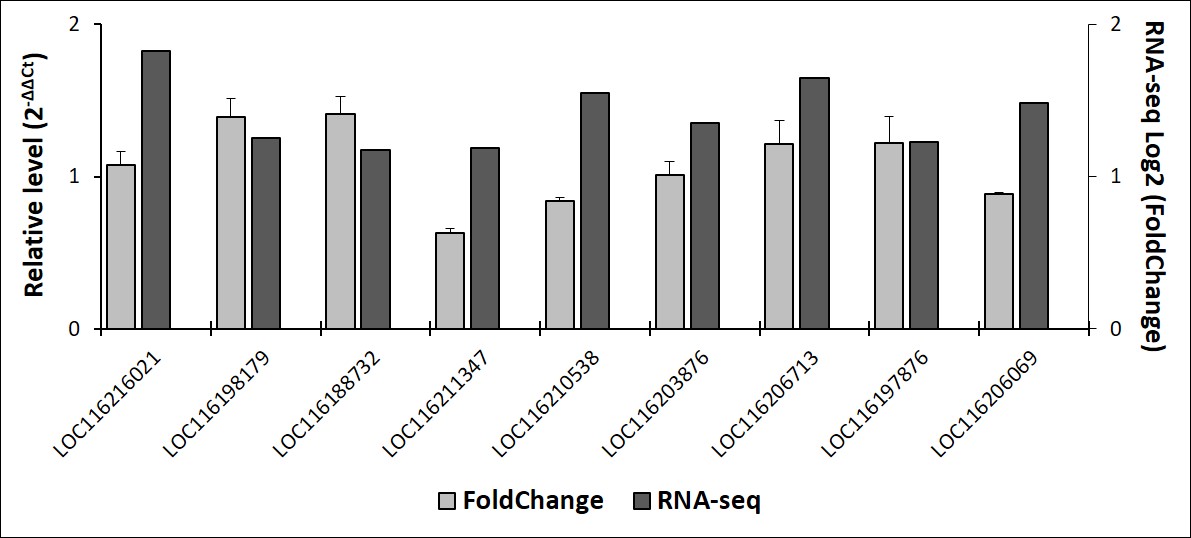

Supplement: Supplementary file 1 [file life-14-00752-s001.zip › Figure S2.jpg]
